# Supplementary material for: Features of Congenital Arthrogryposis Due to Abnormalities in Collagen Homeostasis, a Scoping Review
Source: Int J Mol Sci. 2023 Aug 31;24(17):13545. doi: 10.3390/ijms241713545 (PMC10487887; doi:10.3390/ijms241713545)
Supplement: Supplementary file 1 [file ijms-24-13545-s001.zip › ijms-2534335-supplementary.pdf]

International Journal of *Molecular Sciences*

MDPI

Review

Features of Congenital Arthrogryposis due to Abnormalities in Collagen Homeostasis, a scoping review.

Sarah MacKenzie Picker [1], George Parker [2] and Paul Gissen [3,4,*]

[1] Northumbria Healthcare Trust; sarah.picker98@gmail.com
[2] Newcastle University Medical School; georgeparker6199@gmail.com
[3] National Institute for Health Research great Ormond Street Hospital Biomedical Research Centre, University College London, London, UK.
[4] Genetics and Genomic Medicine Department, Great Ormond Street Institute of Child Health, University College London, London, UK
* Correspondence: p.gissen@ucl.ac.uk

Supplementary Materials

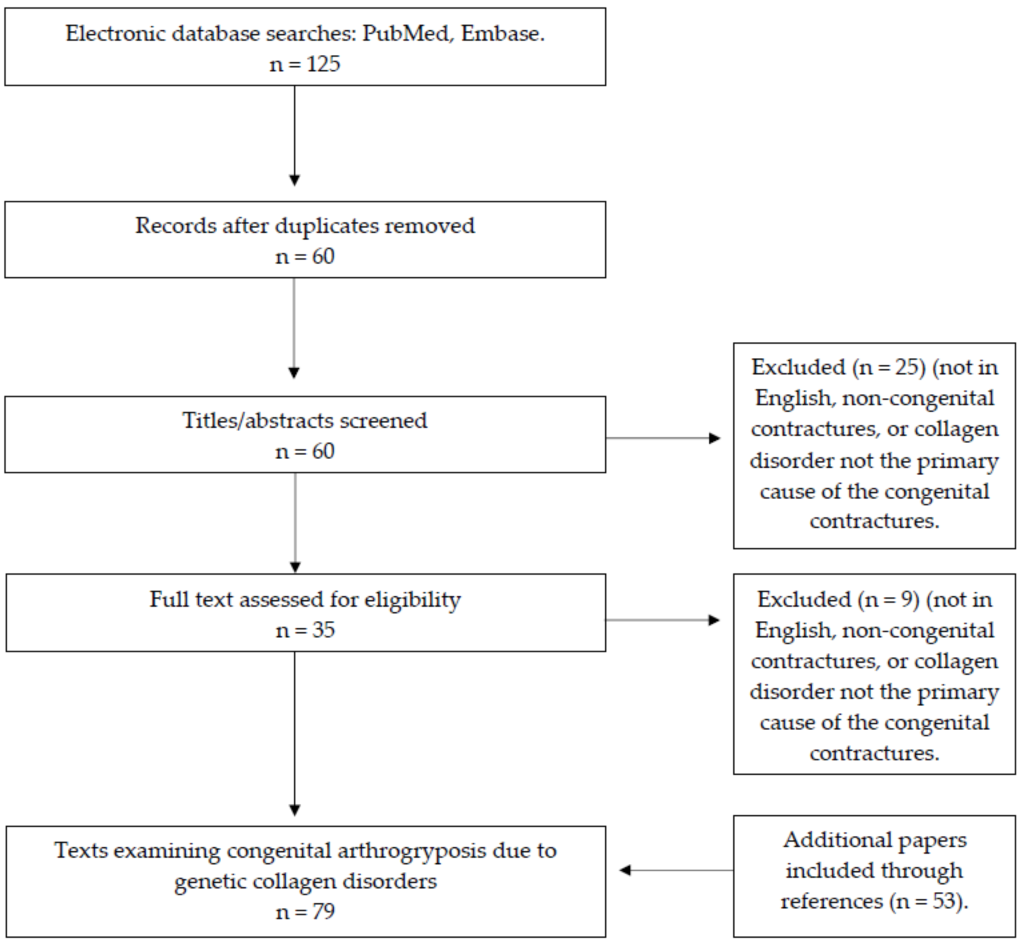

Figure S1. Search strategy flow chart. Flow chart demonstrating the number of papers identified for review, those included and excluded and the reasons for exclusion.
